# Occult Hepatitis B virus infection among HIV negative and positive isolated anti-HBc individuals in eastern Ethiopia

**DOI:** 10.1038/s41598-020-79392-x

**Published:** 2020-12-17

**Authors:** Desalegn Admassu Ayana, A. Mulu, A. Mihret, B. Seyoum, A. Aseffa, R. Howe

**Affiliations:** 1 College of Health and Medical Sciences, Haramaya University, Haramaya, Ethiopia; 2 Armauer Hansen Research Institute, Addis Ababa, Ethiopia

**Keywords:** Microbiology, Diseases, Medical research

## Abstract

The absence of hepatitis B surface antigen (HBsAg) and the presence of antibody to hepatitis B core antigen (anti-HBc) in the blood of apparently healthy individuals may not indicate the absence of circulating hepatitis B virus (HBV) and might be infectious. Despite the risk of HBV transmission, there has been no report from Ethiopia examining this issue; therefore, this study determined occult HBV infection (OBI) among isolated anti-HBc (IAHBc) HIV negative and HIV positive individuals on ART in eastern Ethiopia. A total of 306 IAHBc individuals were included in this study. DNA was extracted, amplified, and detected from plasma using a commercially available RealTime PCR platform (Abbott *m*2000*rt*) following the manufacturer’s instructions. Data were entered into EPI Data version 3.1, cleaned, and analyzed using Stata version 13. Descriptive analysis was used to calculate prevalence, summarize sociodemographic data and other factors. From the 306 IAHBc individuals (184 HIV positive and 122 HIV negative) included in the study, 183 (59.8%) were female of which 142 (77.6%) were within the reproductive age group. DNA extraction, amplified and detection was conducted in 224 individuals. The overall OBI prevalence was 5.8% (5.6% in HIV negative and 6% in HIV positive) among the IAHBc individuals. The HBV DNA concentration among the occult hepatitis B individuals was < 200 IU/mL, indicating a true occult. This study reported the burden of OBI, which pauses a significant public health problem due to the high burden of HBV infection in the country. OBI may cause substantial risk of HBV transmission from blood transfusion, organ transplantation as well as vertical transmission as screening is solely dependent on HBsAg testing.

## Introduction

### Background

Anti-HBc is found in individuals who have experienced natural infection with Hepatitis B virus (HBV), and its presence in the absence of HBsAg is usually interpretable as evidence of past HBV infection^1^. Anti-HBc alone’, referred to as ‘isolated anti-HBc’ (IAHBc) is the presence of anti-HBc in the absence of HBsAg and anti-HBs^1^. Anti-HBc positivity not only provides evidence of prior infections, but also a risk of an ongoing, occult HBV infection, whereby the word “occult” refers to the apparent lack of HBsAg^2^.

Occult blood infection (OBI) was defined as the presence of HBV DNA in liver (with detectable or undetectable HBV DNA in the serum) of HBsAg negative individuals by currently available assays. In resource limited environments, OBI is usually detected by the analysis of serum samples as liver biopsy examinations are not routinely available. When detectable, the amount of HBV DNA in the serum is usually very low (< 200 IU/mL)^3^.

The prevalence of IAHBc and occult HBV infection varies depending on endemicity of Hepatitis B. In low HBV endemic areas, IAHBc is found in 10–20% of all individuals with HBV markers^1^. In the Asia–Pacific Region where HBV is endemic, the prevalence of IAHBc has been reported in up to one-third of the general population^4^. In HBV endemic sub-Saharan countries, the prevalence exceeds over 50% among blood donor population^5^. An overall total anti-HBc prevalence of 32.5% in Nigeria^6^, 7.8% among Egyptian blood donors^7^, 39.1% among HIV positive individuals in Cameroon has been reported^8^. In a recent study, we have reported IAHBc prevalence in one fifth (21%) of the HIV infected individuals on anti-retroviral therapy (ART) in eastern Ethiopia^9^.

A correlation between levels of HBV DNA and HBV seromarkers among patients with OBI were reported^10,11^. HBV DNA level was lowest in patients who were negative for all seromarkers (seronegative patients), intermediate in anti-HBc negative and anti-HBs positive patients, and highest (10–80%) in subjects who were anti-HBc positive but anti-HBs negative (anti-HBc only)^10,11^. This last group is more likely to be infectious, suggesting that anti-HBc does not result in complete HBV elimination^12^.

OBI is related to the long-term persistence of viral covalently closed circular DNA (cccDNA) in the nuclei of hepatocytes^13^. Possible explanations include low-copy numbers of HBV DNA^14^, altered host immune response^15^, genetic variations of the S gene, viral DNA integration in the host genome, infection of peripheral blood mononuclear cells^16^, immune complexes in which HBsAg is hidden^17^, and interference of other viruses such as HCV^18,19^ and HIV^20,21^.

Blood containing anti-HBc without detectable HBsAg might be infectious^22^. Reports indicated evidence of HBV transmission through sexual contact, perinatal transmission^23^ and in blood transfusion^24^ in the absence of HBsAg. A report in some European countries demonstrated 99% sequence homology of HBV DNA in 10 donor-recipient pairs, confirming the infectivity of blood products of OBI carriers^25^.

HBV DNA was detected in about 10% of IAHBc in low HBV endemic areas^1^. Studies of anti-HBc-positive donors have revealed an HBV DNA positivity rate of 0–15%^26^. Among IAHBc individuals, HBV-DNA was detected in serum of 8.1% in Germany^27^, in 1.7% in Korea^28^, in 13% in Lebanon^29^ and in 6.9% of blood donors in Japan^30^. In Africa, an OBI prevalence of 8% in south-eastern Nigeria^31^ and 17% in south-western Nigeria^5^ were reported. Among HIV positive individuals, a prevalence of 5.9%^32^ to 6.9^8^ were reported in Cameroon.

The potential risk for HBV transmission through hemodialysis, blood transfusion and organ transplantation are among some of the clinical implications of OBI. Furthermore, OBI can cause cryptogenic cirrhosis, acute exacerbation, or fulminant hepatitis and development of hepatocellular carcinoma^33^. However, recipient immune status and the number of HBV DNA copies determine the clinical outcome of occult HBV transmission^26^.

Even though there was no OBI report in Ethiopia so far, the HBsAg prevalence ranges from 2.1^34^ to 10.9%^35^ among healthy blood donors, and 2.7^36^ to 11.7%^9,37^ among HIV positive individuals. Ethiopia introduced Hepatitis B vaccine in the form of pentavalent to the national immunization program in March 2007^38^ Though Ethiopia, the second most populous nation in Africa, considered hyperendemic for HBV infection, there is no data that indicates the burden of OBI among IAHBc individuals. Thus, this study aimed to determine the magnitude of OBI among HIV negative and ART experienced HIV positive individuals in eastern Ethiopia.

## Materials and methods

### Study setting and period

The study was conducted in ART clinics and medical Out Patient Departments (OPD) in three selected public hospitals (Hiwot Fana Specialized University Hospital, Dilchora General Hospital, and Karamara General Hospital located in Harar, Dire Dawa and Jigjiga towns, respectively), in eastern Ethiopia. The study was conducted between September 2017 and February 2018.

### Study population

HIV positive individuals on ART were recruited from ART clinic in the three public hospitals. Medical OPD clients were recruited after being tested negative for HIV following the national algorithm. A total of 306 anti-HBc only positive individuals were included for HBV DNA extraction, amplification and quantification study.

### Data collection and processing

#### DNA extraction, amplification and detection

Standard ELISA procedures were followed to detect HBV seromarkers using BIORAD kits (Monolisa HBsAg ULTRA, Monolisa Anti-HBc PLUS, Monolisa Anti-HBs PLUS, BIORAD, France). DNA extraction, amplification and detection were conducted from 200 μl plasma using ABBOTT *m*2000sp and m2000rt System ABBOTT RealTi*m*e PCR (Abbott Molecular Inc.). The target sequence for the Abbott RealTi*m*e HBV assay is in the Surface gene in the HBV genome. This region is specific for HBV and is highly conserved. The primers are designed to hybridize to this region with the fewest possible mismatches among HBV genotypes A through H. Target region is upstream of all Tyrosine-methionine aspartate-aspartate (YMDD), HBsAg, and drug resistant mutants. The amplification cycle at which fluorescent signal is detected by the Abbott *m*2000*rt* is inversely proportional to the log of the HBV DNA concentration present in the original sample^39^. In order to check for consistency, 10% of the samples were retested.

#### Result calculation

The concentration of HBV DNA in a sample or control was calculated from either a stored calibration curve, or a calibration curve created by calibrators within a calibration or sample run. The Abbott *m*2000*rt* instrument automatically reports the results on the *m*2000*rt* workstation. Assay results are reported in IU/mL or Log IU/mL. Results can also be reported in copies/mL or Log copies/mL using an average conversion factor of 3.41 (1 IU = 3.41 copies). The limit of detection of the RealTi*m*e HBV assay is 15 IU/mL with the 0.2 mL sample preparation procedure^39^.

### Quality control

Samples were stored at − 80 °C until processed. Standard operating procedures (SOP) and pre-analytical, analytical and post analytical quality control measures were applied. Enzyme Linked Immuno Sorbent Assay (ELISA) test results were determined based on the cut-off values following the manufacturer’s instruction. Internal control kit with HBV negative, HBV low positive and HBV high positive were used with each run according to the manufacturer’s instruction.

### Data management and analysis

Data were cleaned, coded and entered into EPI Data version 3.1 and analysed using Stata version 13 (StataCorp. 2013. Stata Statistical Software: Release 13. College Station, TX: StataCorp LP). Descriptive analysis was used to calculate prevalence, summarize sociodemographic and other factors.

### Ethical considerations

This study was reviewed and approved by the Institutional Health Research Ethics Review Committee (IHRERC) of the College of Health and Medical Sciences, Haramaya University (Ref. No. IHRERC/137/2017) and AHRI/ALERT Ethics Review Committee, Addis Ababa (Ref. No. P019/17). Written signed informed consent was obtained before data collection. Laboratory results of HBV seromarkers were reported to the respective attending clinician for the necessary intervention. To maintain confidentiality, participants’ information was coded and names and personal identifiers were removed. All methods were carried out in accordance with national research ethics review guideline, Ethiopia^40^. Laboratory analysis was carried out in accordance with the Abbott realtime HBV package insert following manufacturer’s instruction.

## Results

### Socio-demographic characteristics of study participants

From the 306 IAHBc individuals in the study, 184 (60.1%) were HIV positive and 122 (39.9%) were HIV negative, 183 (59.8%) were females of which 142 (77.6%) were within the reproductive age group. The median age of the respondents was 40 years (IQR 32, 46 years) (Table 1).

**Table 1:** Socio-demographic characteristics of IAHBc individuals in public Hospitals, Eastern Ethiopia, 2017/18 (n = 306).

Characteristics	N (%)
**Sex**	
F	183 (59.8)
M	123 (40.2)
**Age (in years)**	
15–21	21 (6.9)
22–34	71 (23.2)
35–49	143 (46.7)
≥ 50	71 (23.2)
**Residence**	
Urban	244 (79.7)
Rural	62 (20.3)
**Family size**	
1–5	241 (78.7)
≥ 6	65 (21.2)
**Marital status**	
Single	54 (17.6)
Married	158 (51.6)
Divorced	94 (30.7)
**Educational status**	
Can’t read and write	95 (31.0)
Read and write	42 (13.7)
Primary and secondary	138 (45.1)
Tertiary	31 (10.1)
**Occupation**	
Government employee	39 (12.7)
Private/self	37 (12.1)
Farmer	29 (9.5)
Student	18 (5.9)
Merchant	33 (10.8)
House wife	58 (19.0)
Daily laborer	38 (12.4)
Unemployed	37 (12.1)
Sex worker	6 (2.0)
Others	11 (3.6)

## Behavioural and health related characteristics of the study participants

From the total IAHBc individuals, 155 (50.7%) had history of body piercing, 61 (19.9%) had tattoo and 30 (9.8%) had history of genital discharge. Furthermore, Khat chewing is a common practice in the area (Table 2).

**Table 2:** Behavioral and health related characteristics of IAHBc individuals in public Hospitals, Eastern Ethiopia, 2017/18 (n = 306).

Characteristics	N (%)
**Alcohol consumption**	
Yes	50 (16.3)
No	256 (83.7)
**Khat chewing**^**a**^	
Yes	88 (28.8)
No	218 (71.2)
**Hospital admission**	
Yes	88 (28.8)
No	218 (71.2)
**Sharp tools injury**	
Yes	25 (8.2)
No	281 (91.8)
**Body piercing**	
Yes	155 (50.7)
No	151 (49.3)
**Tattoo**	
Yes	61 (19.9)
No	245 (80.1)
**Genital discharge**	
Yes	30 (9.8)
No	276 (90.2)
**Surgery**	
Yes	26 (8.5)
No	280 (91.5)
**Tooth extraction**	
Yes	82 (26.8)
No	224 (73.2)
**Share sharp tools**	
Yes	83 (27.1)
No	223 (72.9)
**Emigrated**	
Yes	30 (9.8)
No	276 (90.2)
**HIV status**	
Positive	184 (60.1)
Negative	122 (39.9)

^a^Khat (Catha edulis) is a flowering plant native to Ethiopia. It contains the alkaloid cathinone, a stimulant, which is said to cause excitement, loss of appetite, and euphoria.

### Clinical characteristics of HIV positive IAHBc individuals

All HIV positive IAHBc individuals had been receiving treatment for a median duration of 81.0 months (IQR 45, 115). A total of 170 (93.5%) and 12 (6.5%) were taking first and second line ART regimens, respectively. A total of 121 (66.5%) had ART regimen changed, of which 12 (6.5%) were due to apparent treatment failure. Among IAHBc participants taking second line drugs due to treatment failure, 8 were in WHO clinical stage I category and had good adherence.

Based on the clinical data record of the study participants, 48 (26.1%) had history of tuberculosis (TB) and 103 (56.0%) had a history of opportunistic infections (OI). The vast majority of the participants, 161 (92.5%) had good ART adherence, and 175 (96.7%) were in WHO stage I category (Table 3).

**Table 3:** Clinical characteristics of HIV positive IAHBc individuals on ART in three public hospitals, eastern Ethiopia 2017/18.

Characteristics	n (%)
**History of TB**	
Yes	48 (26.1)
No	136 (73.9)
**History of OI**	
Yes	103 (56.0)
No	81 (44.0)
**Baseline CD4 T cells/mm**^**3**^ **(n = 176)**	
≤ 200	111 (63.1)
201–350	41 (23.3)
351–500	9 (5.1)
≥ 501	15 (8.5)
**Current CD4 T cells/mm**^**3**^** (n = 167)**	
≤ 200	16 (9.6)
201–350	31 (18.6)
351–500	43 (25.7)
≥ 501	77 (46.1)
**Current ART with TDF and 3TC (n = 170)**	
Yes	116 (68.2)
No	54 (31.8)
**Current ART regimen (n = 182)**	
First line	170 (93.5)
Second line	12 (6.5)
**Regimen change**	
Yes	62 (34.1)
No	120 (65.9)
**Reason for regimen change (n = 62)**	
New drug	22 (35.5)
Side effect	20 (32.2)
Treatment failure	12 (19.3)
Others	8 (12.9)
**Duration on ART (n = 184)**	
≤ 6 months	7 (3.8)
> 6 months	177 (96.2)
**WHO clinical stage (n = 183)**	
I	177 (96.7)
II	4 (2.2)
III	1 (0.5)
IV	1 (0.5)
**ART adherence**^**a**^** (n = 179)**	
Good	165 (92.2)
Fair	13 (7.3)
Poor	1 (0.6)

Fair = 85–94% adherence, missed 3–5 doses (of 30 doses), missed 3–9 doses (of 60 doses).

Poor =  < 85% adherence, missed ≥ 6 doses (of 30 doses), missed > 9 doses (of 60 doses).

^a^Good =  > 95% adherence, missed ≤ 2 doses (of 30 doses), missed ≤ 3 doses (of 60 doses).

### HBV DNA among HIV negative and positive IAHBc individuals

From the total of 306 individuals, HBV DNA extraction, amplification and quantification were conducted for 224 (73.2%) individuals, of which 107 (47.7%) were HIV negative and 117 (52.3%) were HIV positive. Of these 224 IAHBc individuals, 211 (94.2%) had no quantifiable plasma HBV DNA, of which 110 (52.1%) were HIV positive individuals on ART. Thirteen individuals (6 HIV negative and 7 HIV positive) had quantifiable HBV DNA in their plasma that make the overall OBI prevalence to be 5.8%. OBI distribution was 5.6% among HIV negative and 6% among HIV positive individuals on ART. The HBV DNA concentration of each IAHBc individuals was < 200 IU/mL.

The mean HBV DNA load in viremic IAHBc individuals was 98.69 IU/mL (SD ± 60.19) among the HIV negative and 62.15 IU/mL (SD ± 60.66) among HIV positives on ART.

Four of the HIV positive OBI individuals were taking ART combinations containing TDF (TDF + 3TC + EFV) for a minimum of 86 months, 4 were female within the reproductive age group and all were in WHO clinical stage I. The rest three were taking AZT + 3TC + NVP for a minimum of 86 months at the time data collection.

## Discussion

This study reported the prevalence of OBI among HIV negative and positive IAHBc individuals in the horn of Africa for the first time. The overall occult hepatitis B infection prevalence among IAHBc individuals was 5.8%. The distribution was 5.6% in HIV negative and 6% in HIV positive individuals on ART. The HBV DNA concentration among the OBI was < 200 IU/mL.

The IAHBc proportion varies greatly depending on the risks of the populations such as blood donors and intravenous drug abusers^1^, gender^27^, immigrants^41^ and HIV infection^9,42^. Studies in Western countries showed that a high proportion of IAHBc had co-infection with HIV or HCV, that could lead to down-regulation or interference with HBsAg production^43,44^.

In HIV co-infected patients, an OBI prevalence of 15% in Ivory Coast^45^, 6.9% in Cameroon^8^, 19.1% in South Africa and 10–15% in Sudan were reported^46,47^. Our study reported a relatively lesser burden of OBI compared to these reports. The variation may depends on a number of factors that include HBV endemicity, liver disease, HBV screening method and primers employed for NAT^5^. Moreover, the true rate in a population may vary because some patients may demonstrate intermittent HBV DNA positivity^48^ which may not be detected in a cross sectional study. OBI is reported to be more common in HIV-positive individuals^49^ and hence known as a risk factor for the development of OBI^21^ due to down-regulation of HBsAg synthesis and surface antigen mutation^50^.

HBsAg negative individuals with serum HBV DNA level < 2.3 log10 IU/mL (< 200 IU/mL), are considered true OBI and individuals with serum HBV DNA level similar to HBsAg positive (“overt”) infection, but are nevertheless HBsAg-negative, have been termed “false” OBI. “False” OBI is usually due to rare infection with *S* gene escape mutants, which produce a modified HBsAg that is not recognized by routinely used detection assays^3,51^. In our study, all the OBI cases have HBV DNA count < 200 IU/mL, indicating true OBI. Almost all OBI cases are infected with replication competent HBV, however, in a small number of OBI cases the low viral load level revealed a strong suppression of replication and gene expression activity due to mutations in the Pol gene or defective synthesis of S proteins due to mutations in the S promoter genomic region^3^.

In this study, four of the OBI individuals in HIV positive group were on ART containing TDF and 3TC for over 6 months. Even though true OBI was indicated regardless of ART, it is also likely that the lower HBV DNA load among HIV positive individuals on ART could be due to drugs such as lamivudine or tenofovir, which often suppress HBV DNA to undetectable levels^52^. HBsAg negativity may also be due to the development of diagnostic escape mutants secondary to the use of ART, as reviewed by Ponde *etal*^53^.

Though the number of HBV DNA positive IAHBc cases seems few in our study, the findings have public health importance because of the possibility of post-transfusion HBV infection in recipients of blood from anti-HBc alone positive donors^54^. In females within reproductive age with OBI, the chance of vertical transmission should not be overlooked during child birth. Research findings indicated Anti-HBc alone is frequently observed in pregnant women^53^. Moreover, anti-HBs concentrations are usually lower and may fall below detection level in individuals who recovered from the infection a long time ago, while anti-HBc persists^1^. In Ethiopia, where HBsAg is the only screening test^9^, problems associated with viral mutations may affect the HBV prevention effort and also the quality of life among HIV positive individuals on ART.

The absence of a close relationship between OBI prevalence and endemicity of HBV infection in our study may indicate the role of other factors. Differences in the population studied, the heterogeneity in the sensitivity and specificity of the methods used and the clinical specimens for the HBVDNA tests^55^ may also affect OBI detection.

In the absence of highly sensitive HBV DNA testing, the use of anti-HBc as a possible surrogate marker for identifying potential seropositive OBI in cases of blood and organ donation^20,56,57^, could be considered as one of the strategies to improve safety among recipients. Furthermore, anti-HBc screening may be a valuable tool to identify subjects previously exposed to HBV and potentially bearing significant risk for HBV reactivation due to immunosuppressive therapies for neoplastic and hematological disorders^57^. The use of HBsAg and anti-HBc screening tests has been the basis of HBV screening in many countries, and this has significantly reduced but did not eliminate transfusion associated HBV (TAHBV)^58^. However, in countries like Ethiopia where the seroprevalence of anti-HBc antibody is quite high, screening leads to rejection of more than a third of the donated blood and may not be applicable for donor selection. Moreover, not all anti-HBc positive subjects are HBV DNA-positive and also the absence of anti-HBc antibody does not exclude sero-negative OBI^3^. OBI can be seronegative (negative for all serological markers), which accounts for approximately 22% of all OBI cases, and seropositive (35% anti-HBs and 42% anti-HBc-positive), which accounts for 78% of OBI^59^. High frequencies of HBV-DNA positivity (10% to 80%) have been observed among anti-HBc only individuals^11,14,60^.

However, in a country where 200,000 units of blood are needed annually for transfusion^61^, and the services are solely based on HBsAg screening, the risk of HBV transmission through OBI has significant public health implications and should be given the necessary attention. In addition, about 52% of blood transfusions made in low-income countries are given to under 5 children^62^. This fact may further increase the risk of HBV transmission and development of chronic hepatitis infection in Ethiopia. This data therefore is useful for policy makers to scale up HBV screening in order to achieve the global health sector strategy (GHSS) of viral hepatitis elimination target to identify 30% of persons living with HBV by 2020, 90% by 2030 and reducing mortality by 65% in 2030^63^. Though the clinical significance of OBI is dependent on many factors^26^, HBV transmission may increase due to the weak immunity of a patient who require hemodialysis, blood transfusion or organ transplant and also the low HBV on birth vaccination coverage in our setting.

## Limitations

This study assessed only the sero-positive OBI individuals due to resource limitations, that may have affected the true prevalence of OBI. Additionally, nucleic acid amplification from peripheral blood may not be sufficiently sensitive because HBV latency within liver cells produces only sporadic HBV DNA in blood. Furthermore, the lack of HBV sequence data from the occult infections may limit the information generated in terms of genetic variability and drug resistance.

## Conclusions and recommendations

This study reveals the potential but unseen risks of HBV transmission due to OBI for the first time in Ethiopia. The IAHBc proportion and the burden of occult hepatitis B have a significant public health implication due to the possibility of HBV transmission through blood and blood products transfusion, organ transplantation and vertical transmission, as screening is solely dependent on HBsAg testing only. This finding may alarm responsible institutions to upgrade such services in order to reduce the risk of HBV transmission from OBI donors.

In addition to HBsAg, detecting HBV DNA among the IAHBc individuals with affordable technology may improve the safety of recipients with a reasonable donation deferral and therefore help the HBV prevention effort in endemic areas.

## Data Availability

All data supporting the results reported in the article are include in the manuscript.
